# Progress in the clinical effects and adverse reactions of ticagrelor

**DOI:** 10.1186/s12959-023-00559-3

**Published:** 2024-01-10

**Authors:** Peng Wei, Xiaoqing Wang, Qiang Fu, Bangming Cao

**Affiliations:** 1 Department of Cardiology, Shanghai Jiao Tong University Affiliated Sixth People’s Hospital, Shanghai, 200233 China; 2 Department of Cardiology, Xuzhou Central Hospital, Xuzhou, 221009 Jiangsu China; 3 Department of Gerontology, The Affiliated Hospital of Youjiang Medical University for Nationalities, No. 18# Zhongshan 2 Road, Baise, 533000 Guangxi Zhuang Autonomous Region China

**Keywords:** Ticagrelor, Acute coronary syndrome, Clinical effect, Adverse reaction

## Abstract

**Background:**

Ticagrelor is a novel receptor antagonist that selectively binds to the P2Y12 receptor, thereby inhibiting adenosine diphosphate (ADP)-mediated platelet aggregation. Compared to clopidogrel, ticagrelor has the advantages of a fast onset, potent effects, and a reversible platelet inhibition function, which make this drug clinically suitable for treating acute coronary syndrome (ACS), especially acute ST-segment elevation myocardial infarction (STEMI).

**Objective:**

This review was performed to determine the basic characteristics, clinical effects, and adverse reactions of ticagrelor.

**Methods:**

Relevant trials and reports were obtained from the MEDLINE, Embase, and Cochrane Library databases.

**Results:**

Ticagrelor is rapidly absorbed by the body after oral administration, exhibits inherent activity without requiring metabolic activation, and binds reversibly to the P2Y12 receptor. Ticagrelor has been recommended in ACS treatment guidelines worldwide due to its advantageous pharmacological properties and significant clinical benefits. Ticagrelor inhibits platelet aggregation, inhibits inflammatory response, enhances adenosine function, and has cardioprotective effects. However, ticagrelor also causes adverse reactions such as bleeding tendency, dyspnea, ventricular pause, gout, kidney damage, and thrombotic thrombocytopenic purpura in clinical treatment. Therefore, it is necessary to pay attention to risk assessments when using ticagrelor.

**Conclusion:**

Ticagrelor is a promising drug for the effective treatment of ACS. When using ticagrelor, individualized treatment should be provided based on the specific conditions of the patients to avoid serious adverse events.

## Introduction

Since its approval in 2010, ticagrelor has been widely used as an antiplatelet agent for the treatment of clinical acute coronary syndrome (ACS). Ticagrelor is a novel P2Y12 receptor antagonist that is rapidly absorbed by the body after oral administration. Therefore, compared to traditional P2Y12 receptor inhibitors such as clopidogrel and prasugrel, ticagrelor has the advantages of a fast onset, high efficacy, and reversible platelet inhibition. Ticagrelor has been recognized worldwide because of its favorable pharmacological properties and significant clinical benefits, and it is the preferred antiplatelet drug for the treatment of ACS [1]. However, in-depth studies and the expansion of the drug’s clinical applications have gradually revealed that ticagrelor also induces various adverse reactions, including bleeding tendency, dyspnea, ventricular pause, gout, kidney damage, and thrombotic thrombocytopenic purpura (TTP), which can limit its use. The basic characteristics, clinical efficacy, and adverse reactions of ticagrelor are reviewed in this article to improve the clinical understanding of this drug. This article is based on previously conducted studies and did not involve human participants or animal experimentation.

## Chemical characteristics and mechanism of ticagrelor

Ticagrelor is not a prodrug and therefore does not require metabolic activation to inhibit the P2Y12 receptor. Ticagrelor is a non-thiophene pyridine adenosine diphosphate (ADP) receptor antagonist that has a chemical structure similar to adenosine and belongs to the cyclopentyltriazolopyrimidine class of antiplatelet drugs [2]. Ticagrelor exerts its antiplatelet effects by suppressing the expression, binding, and activity of P2Y12, an ADP receptor found on platelet membranes.

## Pharmacokinetics and pharmacodynamics of ticagrelor

Effective blood concentrations of ticagrelor can be achieved within 1.5–3.0 h after oral administration. Ticagrelor is then transformed by liver enzymes into active metabolites [3] that synergistically generate antiplatelet effects in vivo [4]. The major metabolites of ticagrelor are AR-C124910XX and AR-C133913XX, which are mainly metabolized by CYP3A4 and CYP3A5 [5]. AR-C124910XX has been identified as a ticagrelor active metabolite (TAM) because its potency in binding P2Y12 is similar to that of ticagrelor. Therefore, it is necessary to neutralize ticagrelor and TAM to effectively reverse the effects of ticagrelor therapy [6]. After repeated metabolism in the liver, ticagrelor and its metabolites are excreted in the feces, with only a small part excreted through the kidneys [7]. Moreover, ticagrelor shows faster, more effective antiplatelet effects than clopidogrel does in patients with chronic renal failure undergoing dialysis, indicating that ticagrelor can be used for antiplatelet therapy in patients with renal insufficiency [8]. The pharmacokinetic characteristics of ticagrelor are not significantly correlated with the patients’ age, gender, or ethnicity and are not affected by diet. The use of ketoconazole, a strong CYP3A4 inhibitor, has been found to overtly increase the mean maximum concentration and mean area under the plasma concentration-time curve of ticagrelor by 135% and 632%, respectively, indicating that the co-administration of ticagrelor and ketoconazole should be avoided [9]. Similarly, the simultaneous use of ticagrelor and other potent CYP3A4 inhibitors, including itraconazole, clarithromycin, ritonavir, and telithromycin, is contraindicated [10]. Opioids, such as fentanyl and morphine, have been demonstrated to impair the antiplatelet effects of ticagrelor, raising the risk of insufficient platelet inhibition and leading to thrombotic complications in patients with coronary artery disease [11, 12]. Opioids may delay the absorption and effects of oral P2Y12 receptor antagonists in patients, resulting in reduced plasma concentrations, delayed antiplatelet action, and increased platelet reactivity [13]. Thus, ticagrelor should also not be taken concomitantly with opioids.

Pharmacodynamic investigations of ticagrelor have revealed its fast, potent, reversible inhibition of platelets. Notably, ticagrelor does not require metabolic activation in the live, as it is quickly activated after absorption, and its efficacy is not affected by liver function [14]. Studies have shown that the binding and dissociation reactions between the ticagrelor and the P2Y12 receptor are rapid and reversible [15]. Interestingly, ticagrelor has been shown to elevate extracellular adenosine levels in vitro by repressing the equilibrative nucleoside transporter 1 (ENT1), which is an adenosine transporter found in red blood cells [16]. Thus, ticagrelor can promote a series of adenosine-induced physiological responses, such as enhanced coronary blood flow and inhibited adenosine-dependent platelet aggregation.

Nonlinear mixed-effects modeling and simulations have been used to determine the exposure–response relationship between ticagrelor and platelet inhibition. In patients with stable coronary artery disease or a history of myocardial infarction (MI), 60 mg or 90 mg of ticagrelor administered twice daily can achieve near-maximal platelet inhibition, whereas doses less than 60 mg lead to weaker overall efficacy and greater differences between patients [17]. Furthermore, evidence has shown that crushed or chewed ticagrelor tablets lead to faster platelet inhibition than standard whole tablets [18]. Although the gastrointestinal absorption of crushed ticagrelor is superior to that of whole tablets, the platelet reactivity of ACS patients is still high in the first two hours after administration, which is a marker of thrombotic complications [19]. Intriguingly, it has been reported that the concomitant administration of cangrelor and crushed ticagrelor can improve the gap in platelet repression that results from administering ticagrelor alone, without any significant drug interactions [20].

## Clinical efficacy of ticagrelor in disease treatment

Ticagrelor has been recommended in ACS treatment guidelines worldwide due to its advantageous pharmacological properties and significant clinical benefits [1, 21, 22]. The clinical therapeutic effects of ticagrelor are mainly attributed to its effective inhibition of the P2Y12 receptor, which inhibits platelet aggregation, suppresses inflammation, reinforces adenosine function, and enhances cardiovascular protection.

### Antiplatelet effects of ticagrelor

The process of intracoronary thrombosis is characterized by the binding of ADP to the P2Y12 receptor on platelet surfaces, which triggers platelet activation. Therefore, the effective inhibition of the P2Y12 receptor has become the main approach to clinically treating coronary artery disease. Ticagrelor reduces thrombosis by selectively inhibiting the P2Y12 receptor, and numerous studies have shown that ticagrelor has potent antiplatelet effects, with a fast onset and small individual differences in effectiveness. Clopidogrel is a P2Y12 inhibitor that is widely used for antiplatelet therapy, but ticagrelor results in faster, stronger effects and a greater reduction of atherothrombotic events in ACS patients [23]. The well-known PLATO study, which involved a 12-month follow-up of patients with ST-segment elevation or non-ST-segment elevation ACS, showed a significantly lower incidence of major cardiovascular events in patients receiving ticagrelor than in those receiving clopidogrel, with no remarkable increase in bleeding risk [24]. In addition, the short-term efficacy of ticagrelor has been reported to be better than that of clopidogrel in patients with ACS: the occurrence of cardiovascular events decreased one month after ticagrelor administration (180 mg loading dose plus 90 mg twice daily maintenance dose), compared to clopidogrel administration (300 mg loading dose plus 75 mg daily maintenance dose); neither ticagrelor nor clopidogrel caused fatal bleeding [25]. More importantly, Liu et al. estimated the antiplatelet function of ticagrelor at different loading doses and found that non-ST-segment elevation ACS patients receiving a high loading dose of 360 mg and a maintenance dose of 90 mg twice daily exhibited faster, stronger platelet suppression than those treated with a conventional loading dose of 180  mg and a maintenance dose of 90 mg twice daily [26]. Compared to clopidogrel, ticagrelor significantly reduces ischemic events without increasing bleeding events in patients undergoing percutaneous coronary intervention (PCI) or coronary artery bypass grafting (CABG), and it also reduces all-cause mortality by 51% among CABG patients [27–29]. Ticagrelor has also been reported to have a faster platelet inhibition rate and a higher degree of inhibition than clopidogrel in diabetic patients with stable coronary heart disease, with the responses being independent of diabetic status. The RESPOND study was the first to evaluate the reactivity of patients with stable coronary heart disease after administration of 300 mg clopidogrel; a crossover experiment based on reactivity showed that the patients’ platelet aggregation decreased from 59% to 35% after switching from clopidogrel to ticagrelor, whereas the it increased from 36% to 56% after switching from ticagrelor to clopidogrel [30]. These results indicate that ticagrelor is a potent alternative to clopidogrel. 

Individuals treated with clopidogrel may exhibit resistance to the drug or variability in clopidogrel efficacy, which may be related to individual differences as well as *CYP2C19* gene polymorphisms. The CYP2C19 loss of function (LoF) allele is associated with a significantly increased risk of major adverse cardiovascular events in patients with stable coronary artery disease undergoing PCI and treated with clopidogrel [31]. However, compared to clopidogrel, ticagrelor significantly reduces the risk of major adverse cardiac events and cardiovascular death among ACS patients carrying the CYP2C19 LoF allele and undergoing PCI [32]. Stimpfle et al. [33] investigated CYP2C19 gene polymorphisms and confirmed that the efficacy of antiplatelet therapy significantly improved in patients with poor responses to clopidogrel after switching to ticagrelor. In addition, ticagrelor has been recommended over clopidogrel for ACS patients with ABCB1 gene polymorphisms [34]. In sum, ticagrelor is not affected by individual genetic polymorphisms, so it can be used for the treatment of clopidogrel-resistant patients. Of note, the CYP3A4*22 allele has been shown to significantly attenuate the elimination of ticagrelor, thereby enhancing its antiplatelet effect [35]. However, a study in a Chinese population have indicated that it is unnecessary to adjust the dose of ticagrelor according to the CYP3A4 genotype [36].

### Anti-inflammatory effects of ticagrelor

Platelets not only participate in hemostasis and thrombosis but are also involved in the body’s inflammatory response. Antiplatelet drugs can inhibit platelet aggregation and suppress inflammation. The P2Y12 receptor is expressed on the surfaces of platelets and various inflammatory cells (e.g., neutrophils, dendritic cells, and monocyte-macrophages), so the activation of the P2Y12 receptor plays a key role in the activation and migration of inflammatory cells [37, 38]. In early animal experiments, clopidogrel significantly delayed the occurrence and development of mouse aneurysms by reducing the expression of inflammatory factors and the infiltration of inflammatory cells [39]. Further, the ASCET study showed that blood levels of C-reactive protein (CRP), tumor necrosis factor (TNF), interleukin (IL), matrix metalloproteinases (MMPs), and cluster of differentiation 40 (CD40) ligands were markedly reduced in patients with coronary heart disease after one year of clopidogrel treatment [40]. Chen et al. [41] found lower high-sensitivity CRP (hs-CRP) concentrations in patients with non-ST-segment elevation ACS who were treated with a combination of clopidogrel and aspirin than in patients treated with aspirin alone. In a recent study [42], ticagrelor treatment resulted in a great reduction of hs-CRP compared to clopidogrel, suggesting that ticagrelor has a stronger anti-inflammatory effect. The mechanism by which ticagrelor exerts its anti-inflammatory effect is still unclear, but it probably acts by blocking the formation of platelet-macrophage and platelet-neutrophil aggregates. Gasecka et al. [43] reported that the fragments released by activated platelets during ACS are platelet-derived extracellular vesicles (PEVs); they are likely to be involved in inflammation and thrombosis and potentially in the development and progression of atherosclerosis. PEVs released by activated platelets (positive for CD61, P-selectin, and phosphatidylserine) are referred to as PEVs+. Notably, ticagrelor has been found to attenuate inflammation and thrombosis by decreasing the concentration of PEVs+.

### Adenosine-like effects of ticagrelor

Ticagrelor can inhibit the uptake of adenosine by red blood cells, increase the concentration of extracellular adenosine, and enhance the activity of adenosine to produce a series of biological effects, including platelet inhibition, vasodilation, and myocardial protection [44]. Van Giezen et al. [45] reported that this inhibitory effect of ticagrelor on adenosine uptake could result in increased extracellular adenosine concentrations. In subsequent animal experiments, ticagrelor and dipyridamole were found to significantly and dose-dependently increase intracoronary adenosine-mediated blood flow in open-chest canine models. Wittfeldt et al. [46] obtained similar results from clinical randomized double-blind trials: ticagrelor significantly and dose-dependently increased the coronary blood flow velocity and area under the velocity curve when compared with a placebo. Similarly, in a prospective, single-center, single-blind study by Alexopoulos et al. [47], the ticagrelor group exhibited a significant increase in coronary blood flow velocity compared to the prasugrel group. Taken together, these findings suggest that ticagrelor plays an active role in the adenosine-mediated increases in coronary blood flow.

Animal experiments conducted by Wang et al. [48] and Birnbaum et al. [49] revealed that ticagrelor has a unique cardioprotective mechanism that clopidogrel does not. H-FABP can be used to determine myocardial infarct size in patients with ST-segment elevation myocardial infarction (STEMI) and to evaluate their cardiovascular prognoses. Studies have shown that the increased levels of hs-TnT and h-FABP after PCI are significantly lower with ticagrelor administration than with clopidogrel administration, thus proving the myocardial protective effect of ticagrelor [50]. Compared to clopidogrel, pretreatment with a loading dose of ticagrelor can significantly reduce the incidence of perioperative MI associated with PCI. A multivariate analysis revealed a negative correlation between ticagrelor use and perioperative MI associated with PCI, indicating that ticagrelor treatment is an independent protective predictor of perioperative MI [51]. This cardioprotective effect is mediated by adenosine and can be completely reversed by adenosine receptor antagonists. Ticagrelor has been found to increases adenosine levels in two ways: 1) by blocking human equilibrium nucleoside transporters to inhibit adenosine reuptake; and 2) through the increased release of adenosine triphosphate (ATP), which is then converted to adenosine [52]. In short, ticagrelor not only inhibits adenosine reuptake but also induces human red blood cells to release ATP in a dose-dependent manner. The ATP released is rapidly decomposed into adenosine by the enzymes in endothelial cells, white blood cells, and red blood cells, which thereby causes transient ventricular block and endothelium-dependent vasodilation [53]. However, increased adenosine levels can cause some adverse reactions, such as dyspnea, bradycardia, gout, kidney damage, and TTP. For example, by acting on the A1 and A2A receptors on the C fibers of the vagus nerve, adenosine provokes bronchial constriction and eventually leads to dyspnea [54]. Bradycardia and ventricular pauses are side effects of adenosine-related stimulation of the adenosine A1 receptor present in heart tissue [55].

## Ticagrelor-related adverse reactions

Numerous ACS patients now benefit from the advantageous antiplatelet effects of ticagrelor. However, due to the pharmacological properties and increasing clinical application of ticagrelor, some adverse reactions of using this drug in antiplatelet therapy, including bleeding tendency, dyspnea, ventricular pause, gout, kidney damage, and TTP, are now receiving considerable attention as part of efforts to make timely adjustments to ensure the safety and maximum clinical benefits of ticagrelor. Moreover, the PROGRESS trial has suggested that, compared to telephone follow-ups, clinical follow-ups in a dedicated outpatient clinic contribute to the decreased incidence of adverse reactions caused by ticagrelor [56]. Notably, a recent study showed that low doses of ticagrelor are safe for long-term antiplatelet therapy, and appropriate patient selection can help avoid or reduce the occurrence rates of some adverse effects of ticagrelor [57].

### Bleeding tendency

Ticagrelor reduces the risk of ischemia but increases the tendency to hemorrhage. Bleeding is the most significant adverse reaction that occurs during the use of antiplatelet drugs, leading to complications, death, and increased healthcare costs. It is also more difficult to stop bleeding during events such as injury or surgery in patients taking ticagrelor due to the inhibition of platelet activity. The specific bleeding events associated with ticagrelor include nose bleeding, subcutaneous hemorrhage, gastrointestinal bleeding, and intracranial hemorrhage. The DISPERS study demonstrated an increased risk of minor bleeding but few major bleeding events with ticagrelor (administered at 50, 100, or 200 mg twice daily)  compared to clopidogrel (administered at 400 mg daily) [58]. The PLATO study showed no significant difference in terms of major bleeding between the ticagrelor group (180 mg loading dose, 90 mg loading dose twice daily thereafter) and the clopidogrel group (300-600 mg loading dose, 75 mg loading dose twice daily thereafter) ; however, major bleeding not related to coronary-artery bypass grafting were higher in the ticagrelor group than in the clopidogrel group, and ticagrelor resulted in an increased risk of intracranial haemorrhage, with a 10-fold increase in the incidence of fatal intracranial hemorrhage (0.1% vs. 0.01%) [24]. For STEMI patients aged under 75 years treated with fibrinolytic therapy in TREAT trial, the rates of major bleeding (1.6% vs 2.1%; P = 0.21), Bleeding Academic Research Consortium (BARC) types 3 to 5 bleeding (1.6% vs 2.0%; P = 0.43), fatal bleeding (0.3% vs 0.2%; P = 0.55), and intracranial bleeding (0.3% vs 0.2%; P = 0.76) were similar between the ticagrelor and the clopidogrel groups, respectively [59]. A meta-analysis was recently conducted to assess the efficacy and safety of ticagrelor in Asian patients with ACS in real-world practice, as compared to clopidogrel; the findings indicated that ticagrelor can reduce the risk of major adverse cardiac events by reducing the risk of stroke, without increasing the rates of major bleeding [60].

However, compared with clopidogrel, ticagrelor is associated with a significantly high incidence of clinically relevant bleeding complications as part of dual or triple antithrombotic therapy. It has been found that the combination of aspirin and ticagrelor (n = 5853) is superior to aspirin alone (n = 10,722) in preventing stroke recurrence or death within 90 days of treatment initiation, but it increases the risk of major bleeding [61]. In patients receiving triple therapy (TT), ticagrelor may increase thromboembolic and ischemic cardiac events [62]. In a systematic review analyzing 5,659 patients who underwent dual antiplatelet therapy or TT, the use of ticagrelor was associated with a higher rate of clinically significant bleeding than clopidogrel was [62]. In addition, the administration of ticagrelor in TT is related to an increased risk of intracranial bleeding, as well as complications such as neurological deficits [63, 64]. Considering this, bridge anticoagulation should be applied with caution when ticagrelor is used in dual antiplatelet therapy or TT.

In TWILIGHT, a double-blind trial, patients with an ischemic event who underwent PCI and completed three months of dual antiplatelet therapy were considered, and ticagrelor monotherapy was associated with a lower incidence of clinically relevant bleeding than ticagrelor plus aspirin. Further, the incidence of the primary endpoint BARC type 2, 3, or 5 bleeding was 4.0% among patients randomly assigned to receive ticagrelor plus placebo and 7.1% among patients assigned to receive ticagrelor plus aspirin (hazard ratio = 0.56; 95% CI = 0.45–0.68; *P* < 0.001) [65].

Platelet transfusion is usually performed for bleeding caused by antiplatelet drugs, but this may not be effective for ticagrelor-caused bleeding. According to a report, the use of 17 units of platelets failed to counteract bleeding due to tegretol in a 65-year-old male emergency patient [66]. Martin et al. [67] found that an increase in platelets could not restore the platelet aggregation function in ticagrelor samples, perhaps because ticagrelor and its metabolites inhibit fresh platelets and thereby prevent infused platelets from achieving a good antibleeding effect against ticagrelor. Notably, antibody fragments that exhibit high-affinity binding to ticagrelor, including MEDI2452 and PB2452, can act as antidotes in reversing the antiplatelet effects of ticagrelor [68, 69]. Antiplatelet therapy is thus a double-edged sword, with an increase in thrombotic events in low responders and an increased risk of bleeding in high responders. Therefore, clinical medication should be differentiated, the risk of ischemia and bleeding should be assessed, and ticagrelor doses should be appropriately adjusted to avoid administering large doses to people with a high risk of bleeding. The importance of individualized treatment should be emphasized when striving for maximum clinical benefit and a minimal risk of bleeding. The ongoing POPular AGE test by Qaderdan et al. [70] is an evaluation of the clinical net benefits of new antiplatelet drugs, and the results may provide further references for clinical drug selection for the elderly.

### Dyspnea

Dyspnea is the most common adverse reaction associated with the clinical use of ticagrelor. The PLATO study showed that dyspnea is likelier to develop in patients treated with ticagrelor than in those treated with clopidogrel (13.8% vs. 7.8%, *P* < 0.001) [24]. Furthermore, Caldeira et al. [71] reported that ticagrelor causes a higher incidence of dyspnea than clopidogrel or prasugrel. Dyspnea tends to be mild to moderate in most patients, often occurs in the early stages of treatment, lasts from days to weeks, is generally self-limiting, and has no significant effects on blood oxygen saturation, vital capacity, functional residual capacity, or diffusion function [72]. Most patients continue to take medication, and their symptoms are gradually relieved without targeted treatment; the symptoms of dyspnea disappear after drug withdrawal [73]. At present, the pathogenesis of dyspnea caused by ticagrelor is unclear. The main mechanisms may be related to the delay in adenosine metabolism caused by the ticagrelor-induced inhibition of adenosine uptake by erythrocytes and the delayed adenosine clearance [74]. Ticagrelor can significantly increase coronary blood flow [45] and coronary blood flow velocity [46] by restraining adenosine uptake, which enhances adenosine-induced dyspnea. This dyspnea symptom can be reversed by theophylline drugs [75]. Other researchers have speculated that dyspnea is correlated with the inhibition ofP2Y12 in neuronal cells.

Compared to clopidogrel, ticagrelor treatment has been found to increase the risk of dyspnea in patients with asthma and chronic obstructive pulmonary disease (COPD) [76]. Therefore, ticagrelor should be used with caution when treating patients with asthma and COPD. Studies have also shown that high doses of ticagrelor remarkably increase the probability of dyspnea [77], which suggests that dyspnea is ticagrelor dose-related. Consequently, reducing the dose might reduce or relieve the symptoms of dyspnea, and the appropriate dose should be determined according to the actual situation of each patient.

### Ventricular pause

The ventricular pause associated with ticagrelor mostly occurs at night, for short durations, and with mild symptoms [78]. The PLATO trial revealed a significant increase in the incidence of ventricular pauses in patients receiving ticagrelor, as compared to clopidogrel, but no statistical difference was noted after one month of treatment [24]. Nicol et al. [79] reported a case of ventricular pause caused by oral ticagrelor therapy for nonsustained tachycardia, and Scirica et al. [80] found a significantly greater proportion of ventricular pauses among patients treated with ticagrelor (5.8%) than those treated with clopidogrel (3.6%) in the first week after randomization. Furthermore, some adverse reactions, such as atrioventricular block and atrial fibrillation, have been reported due to the use of ticagrelor [81–83].

The mechanism of ventricular pause induced by ticagrelor remains to be further explored. Some scholars have supported the understanding that P2Y12 inhibitors directly affect the self-discipline or conductivity of the heart [80], while others have suggested a relationship between the occurrence of early ventricular pauses and changes in adenosine metabolism in the body [44, 45, 47]. The increased adenosine concentrations in the sinoatrial node and atrioventricular compartment may aggravate vagus nerve-mediated nocturnal bradycardia, thereby explaining the nocturnal pause caused by ticagrelor.

### Gout

Gout is a possible adverse reaction to long-term treatment with ticagrelor. In the PLATO study in 2009, plasma uric acid concentration increased by 14% after ticagrelor administration for 1–2 months and decreased significantly after one month of withdrawal [24]. Further, Butler et al. found that serum uric acid increased significantly after five days of ticagrelor treatment, although no adverse reactions were caused by elevated uric acid. In recent study, patients undergoing long-term treatment with ticagrelor were twice as likely to develop gout as those in the placebo group [84].

The specific mechanism governing gout may be as follows: first, ticagrelor is metabolized by the kidney, with ticagrelor and its active metabolites inhibiting urate transporter-1 and organic cation transporter-1 [85], both of which play important roles in the absorption and reacceptance of uric acid [86, 87]. This affects the metabolism of uric acid in the kidney and increases the chance of uric acid exposure. Second, ticagrelor inhibits the equilibrium type nucleoside transporters and suppresses the absorption of adenosine [88], resulting in an increased concentration of adenosine. Uric acid is a metabolite of adenosine and adenosine can cause ischemia, hypoxia, and inflammation, eventually leading to hyperuricemia and gout [89].

Ticagrelor-related gout is easily overlooked during long-term medication. Clinicians should regularly check patients’ plasma uric acid concentration and watch out for the symptoms of arthritis and gout caused by elevated uric acid. Clinically, uric acid abnormalities caused by medication alone can be treated in accordance with the protocols for drug-induced hyperuricemia, along with drug replacement. Patients with ACS and hyperuricemia can receive prophylactic treatment and intensive uric acid-lowering therapy in the early stages of taking ticagrelor to prevent drug-related adverse events.

### Kidney damage

Clopidogrel exhibits low reactivity in patients with chronic kidney disease [90, 91]; therefore, ticagrelor treatment is more advantageous in patients with impaired renal function [27]. However, studies have shown that patients treated with ticagrelor have a slightly higher risk of renal adverse reactions than those treated with clopidogrel. Clinically, the creatinine levels of some patients who received ticagrelor were found to be slightly higher than the levels before administration [5, 24, 92]. Van Vuren et al. reported that a patient with rhabdomyolysis due to elevated serum concentrations of rosuvastatin as a result of worsening renal function due to ticagrelor [93]. In the PLATO study [94], the plasma creatinine concentration increased by more than 30% in about a quarter of the ticagrelor-treated patients, and by more than 50% in some ticagrelor-treated patients. The mechanism of action underlying the increase in creatinine concentration is not clear, but it may be related to the extension of the adenosine half-life and the alteration of adenosine metabolism by ticagrelor. Enhanced adenosine concentration reduces glomerular filtration pressure and changes renal blood flow, resulting in elevated creatinine levels. At the same time, creatinine concentration increases with the elevation of uric acid (an adenosine metabolite) in the early stages of ticagrelor treatment [78]. Although adjusting the ticagrelor dose in response to elevated creatinine levels is not recommended in clinical applications, the guidelines of the European Medicines Agency state that patients should be tested for kidney function within one month of the initial use of ticagrelor. In patients with additional risk factors, creatinine concentration should be promptly determined to evaluate the long-term effects of ticagrelor on renal function.

### TTP

TTP is a rare and lethal disorder that has been reported to be correlated with clopidogrel. FDA database results revealed that clopidogrel is the most common agent related to TTP, and the incidence of clopidogrel-associated TTP is four times higher than that of idiopathic TTP, indicating a causal-and-effect relationship [95]. Bennett et al. [96] studied the incidence of TTP in 11 patients during or soon after clopidogrel therapy and reported the possibility of TTP occurring at the beginning of clopidogrel treatment. Ticagrelor has not previously been correlated with TTP, but recent studies have indicated that TTP may be a rare adverse reaction to the drug. Doğan et al. [97] identified a case of ticagrelor-related TTP after the first month of ticagrelor use. Further, Wang et al. [98] reported that a patient with STEMI who incurred ticagrelor-induced TTP after two months of treatment with ticagrelor recovered quickly due to plasmapheresis and then died when ticagrelor was reused; this suggests that ticagrelor might be a contributor to TTP.

## Conclusion

Ticagrelor is a novel P2Y12 receptor inhibitor that has many advantages in antiplatelet therapy. ACS patients can benefit from a series of biological effects resulting from the increased adenosine concentration induced by ticagrelor. However, adverse reactions can arise during clinical treatment, such as bleeding tendency, dyspnea, ventricular pause, gout, kidney damage, and TTP. Therefore, to avoid serious adverse events, individualized treatment should be provided based on the specific conditions of each patient when using ticagrelor. Based on the results of the ISAR-Reaction V randomized trial, the European Society of Cardiology (ESC) guidelines recommend that prasugrel be prioritized over ticagrelor for patients with non-ST segment elevation ACS undergoing PCI. However, the findings of this research are influenced by a bias that is difficult to estimate owing to various reasons. Moreover, studies have shown that the use of ticagrelor was related to improved outcomes compared with clopidogrel and prasugrel [99]. Further studies are needed to determine the prioritization of ticagrelor and prasugrel.

## Data Availability

Data sharing is not applicable to this article as no new data were created or analyzed in this study.
